# Advances in therapeutic oligonucleotide conjugates

**DOI:** 10.1186/s41120-026-00209-8

**Published:** 2026-11-02

**Authors:** Ya Ying Zheng, Yonger Xue, Xucheng Hou, Yizhou Dong

**Affiliations:** Department of Immunology and Immunotherapy, Windreich Department of Artificial Intelligence and Human Health, Department of Oncological Sciences, Icahn Genomics Institute, Charles Bronfman Institute for Personalized Medicine, Precision Immunology Institute, Tisch Cancer Center, Biomedical Engineering and Imaging Institute, Friedman Brain Institute, Icahn School of Medicine at Mount Sinai, New York, NY 10029 USA

**Keywords:** Oligonucleotide therapeutics, Antisense oligonucleotides (ASOs), Small interfering RNAs (siRNAs), Triantennary *N*-acetylgalactosamine (GalNAc), Cell-penetrating peptides (CPPs), Antibody-oligonucleotide conjugates (AOCs), Aptamers

## Abstract

Oligonucleotide therapeutics (ONTs) have revolutionized the medical landscape by enabling the precise modulation of genetic pathways. This perspective highlights the major conjugation strategies for ONTs, using antisense oligonucleotides (ASOs) and small interfering RNAs (siRNAs) as representative modalities. We describe diverse classes of targeting ligands, including small molecules, cell-penetrating peptides (CPPs), antibodies, and aptamers. For example, triantennary *N*-acetylgalactosamine (GalNAc) conjugation has enabled a number of approved oligonucleotide therapeutics. Meanwhile, other emerging conjugation technologies have expanded oligonucleotide delivery to extrahepatic tissues to address a broader range of diseases. We further summarize the clinical translation of these delivery platforms, highlighting approved therapeutics alongside emerging clinical candidates. Finally, we provide an outlook on challenges and opportunities for the next generation of oligonucleotide therapeutics.

## Introduction

ONTs have emerged as an important class of genetic medicine, providing a powerful strategy to modulate disease causing genes that were previously considered undruggable (Roberts et al. 2020, Kulkarni et al. 2021, Christensen et al. 2025). This rapidly expanding therapeutic class includes ASOs, siRNAs, microRNAs, aptamers, and other engineered nucleic acid platforms (Liu et al. 2025). Among these, ASOs and siRNAs have emerged as the most clinically advanced modalities. Both achieve highly selective modulation of disease associated transcripts through complementary RNA recognition but employ distinct mechanism (Khvorova and Watts 2017, Paunovska et al. 2022). ASOs are single-stranded oligonucleotides that regulate gene expression through RNase H-mediated mRNA degradation, splice modulation, or steric blockade of RNA processing and translation (Bennett and Swayze 2010). In contrast, siRNAs are double-stranded oligonucleotides that induce RNA interference by loading the antisense (guide) strand into the RNA-induced silencing complex (RISC), where AGO2 directs sequence specific cleavage of complementary target mRNA. The early therapeutics such as ASO nusinersen for spinal muscular atrophy (SMA), and the lipid nanoparticle formulated siRNA patisiran for hereditary transthyretin mediated amyloidosis (hATTR), established the viability of genetic medicine (Crooke et al. 2021, Jadhav et al. 2024). Despite these progresses, the broader clinical translation of ASO- and siRNA-based therapeutics remains limited by several challenges, including safety, efficacy, pharmacokinetics, and pharmacodynamics. Most critically, efficient and selective delivery to target tissues and cell types continues to be a major barrier (Corey et al. 2022). Inefficient delivery and off-target effects compromise therapeutic potency and frequently result in dose limiting toxicities, restricting broad clinical use (Roberts et al. 2020, Kulkarni et al. 2021, Setten et al. 2019, Egli and Manoharan 2023).

Alongside advances in sequence design, chemical modification has substantially improved the pharmaceutical properties of ASOs and siRNAs (Deleavey and Damha 2012). Modifications to the phosphate backbone, ribose sugar, and nucleobases including phosphorothioate (PS), 2′-*O*-methyl (2′-OMe), 2′-*O*-methoxyethyl (2′-MOE), 2′-fluoro (2′-F), locked nucleic acid (LNA), and constrained ethyl (cEt) chemistries have enhanced nuclease resistance, target affinity, in vivo durability and biodistribution profiles while reducing immunostimulatory effects (Egli and Manoharan 2023, Hofman and Corey 2024, Yin et al. 2019). These chemical modifications now form the foundation of many oligonucleotide therapeutics; however, they alone are often insufficient to overcome the biological barriers associated with tissue specific delivery.

To address these challenges, conjugation strategies have proven to be a critical approach for achieving selective tissue and cell targeting while improving intracellular delivery. These strategies have further expanded the clinical potential of oligonucleotide therapeutics by increasing efficacy while preserving key therapeutic attributes, such as prolonged stability and low immunogenicity. This perspective summarizes current progress in ASO and siRNA therapeutics, with particular emphasis on conjugation based strategies that overcome delivery limitations and expand the therapeutic reach of ONTs. By integrating rational chemical design with targeted delivery principles, these innovations pave the way toward more precise, effective, and personalized oligonucleotide therapeutics.

### Conjugation strategies for oligonucleotides

**Fig. 1:**
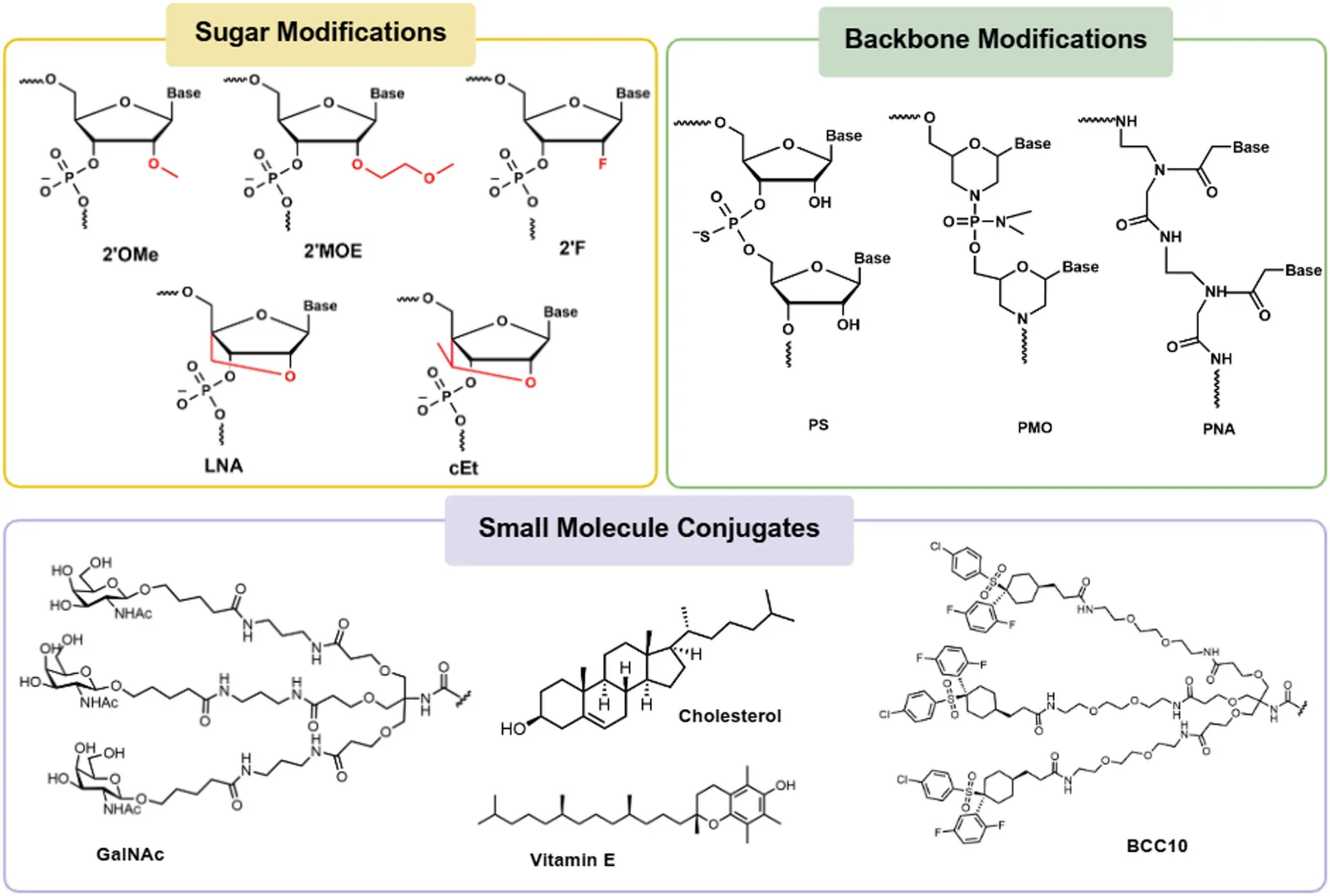
Chemical modifications and structure of small molecule conjugates. Common sugar modifications include 2′-*O*-methyl (2′-OMe), 2′-*O*-methoxyethyl (2′-MOE), 2′-fluoro (2′-F), locked nucleic acid (LNA), and constrained ethyl (cEt). Backbone modifications and alternative scaffolds, including phosphorothioate (PS), phosphorodiamidate morpholino oligomer (PMO), and peptide nucleic acid (PNA). The bottom panel provides representative small molecule ligands used for delivery of oligonucleotide therapeutics

**Fig. 2:**
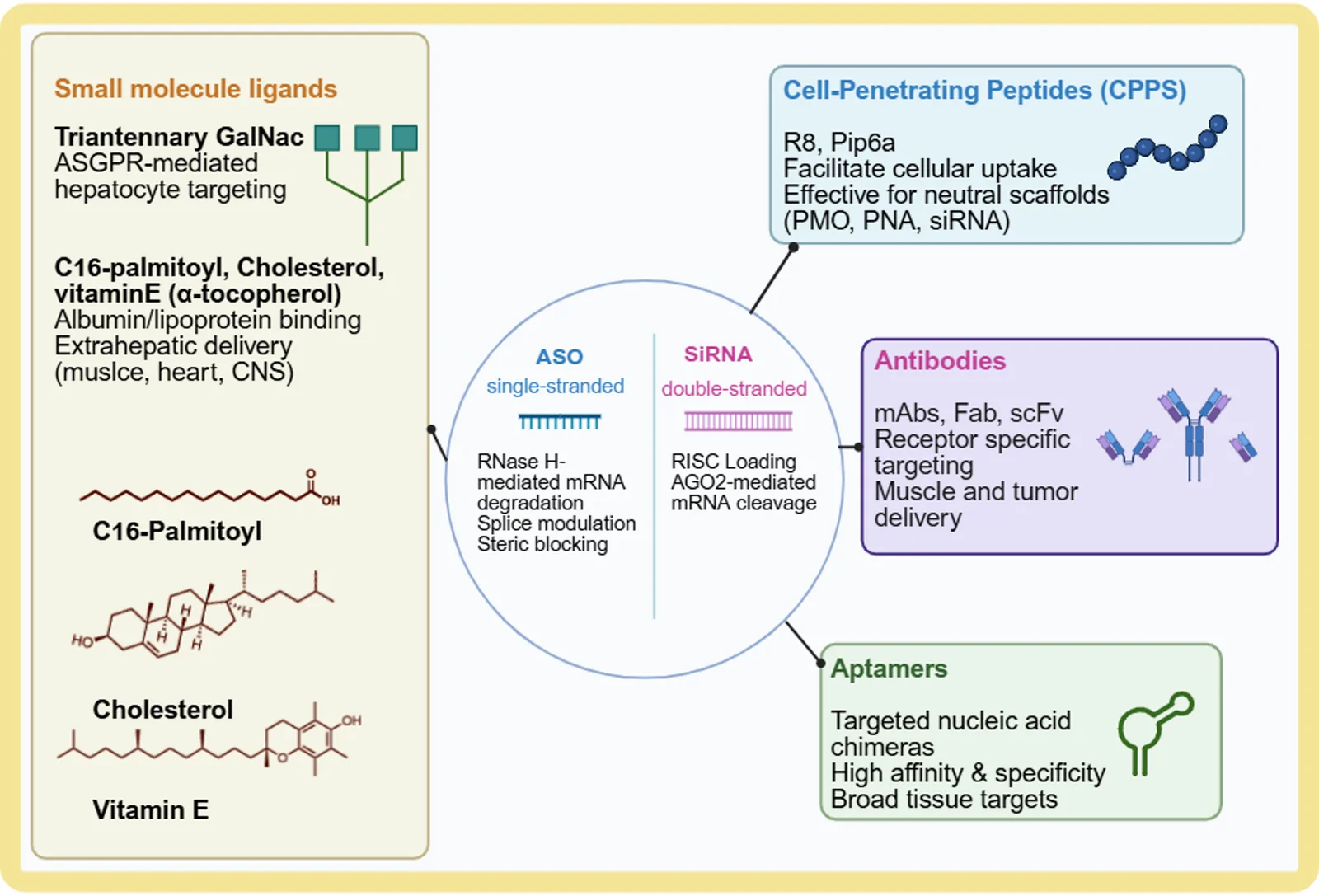
Representative ligand platforms for oligonucleotide delivery. ASOs and siRNAs can be covalently conjugated to diverse ligands to improve cellular uptake, tissue distribution, and therapeutic delivery. Small molecule ligands include GalNAc for ASGPR-mediated hepatocyte targeting and lipophilic moieties, including C16-palmitoyl, cholesterol and vitamin E for protein-associated transport and tissue uptake. Complementary approaches include cell-penetrating peptides (CPPs) promote intracellular delivery through membrane interaction and endocytosis, aptamers for high affinity molecular recognition, and antibody-oligonucleotide conjugates (AOCs) for receptor specific targeting. Together, these diverse ligand platforms expand the tissue specificity and therapeutic potential reach of ONTs, providing a versatile toolkit for the rational design of targeted genetic medicines

Covalent ligand conjugation has emerged as a critical strategy for advancing ONTs toward clinical translation. Figure 1 summarizes key chemical modifications used to enhance oligonucleotide stability and pharmacological properties, together with the structures of representative small molecule ligands for targeted delivery. Ligand identity and linker directly influence pharmacokinetics, biodistribution, receptor engagement and intracellular trafficking (Roberts et al. 2020, Winkler 2013, Juliano et al. 2014, Juliano 2016). For example, small molecule ligands have achieved key clinical milestones. In particular, GalNAc has become the benchmark platform for hepatocyte specific delivery (Corey et al. 2022). GalNAc exhibits high affinity binding to the asialoglycoprotein receptor (ASGPR) on hepatocytes, facilitating efficient receptor mediated endocytosis (Debacker et al. 2020). Clinically, this platform is validated by multiple FDA-approved GalNAc-siRNA therapeutics including inclisiran, givosiran, nedosiran, lumasiran, and vutrisiran, as well as ASO-based eplontersen and donidalorsen. Many other GalNAc conjugated candidates are currently under clinical evaluation (Sun et al. 2024, Tang and Khvorova 2024). These GalNAc conjugated therapeutics use different designs, with GalNAc ligands attached at the 3’ or 5′ end or at other positions within the oligonucleotide. Studies have shown that the number and arrangement of GalNAc ligands, as well as linker length and structure, can strongly influence ASGPR binding, hepatocyte uptake, and gene silencing activity. Most clinically developed GalNAc conjugates use a triantennary design to preserve nuclease stability and RISC- or RNase H-mediated activity while maximizing ASGPR engagement through multivalent receptor interactions. Thus, optimizing ligand number, placement, and linker is important for achieving effective hepatocyte targeting while maintaining oligonucleotide stability and activity (Das et al. 2025, Matsuda et al. 2015) (Fig. 2).

In parallel, ligands such as C16-palmitoyl acid, cholesterol, and α-tocopherol (vitamin E) promote membrane interactions and facilitate engagement with endogenous physiological pathway, including plasma protein binding and lipoprotein mediated transport (Raouane et al. 2012, Biscans et al. 2019). Palmitic acid-ASOs, interact with serum albumin, which promotes cellular internalization via caveolin dependent endocytosis (Chappell et al. 2020). This modification has proven particularly effective for achieving potent silencing in skeletal muscle (Prakash et al. 2019) and enables significant therapeutic potency in oncology. For instance, the palmitoylated-ASO GRN163L, demonstrates marked telomerase inhibition across multiple solid tumor types, including glioblastoma (Marian et al. 2010), hepatoma (Djojosubroto et al. 2005), breast (Gellert et al. 2006), and lung cancers (Dikmen et al. 2005). siRNAs bearing a 2′-O-hexadecyl (C16-palmitoyl) modification achieve effective delivery to the central nervous system (CNS), eye, and lung with durable target suppression and a favorable safety profile (Brown et al. 2022). Lipophilic moieties like cholesterol and α-tocopherol are primarily utilized to facilitate preferential hepatic accumulation via endogenous lipoprotein pathways. Cholesterol-ASO conjugates interact with both high-density lipoprotein (HDL) and low-density lipoprotein (LDL), particles, whereas α-tocopherol-ASOs primarily associate with HDL. The subsequent cellular internalization of these complexes is mediated by specific lipoprotein receptors, notably the scavenger receptor B type 1 (SR-BI) (Ostergaard et al. 2019). This enhanced hepatotropism has established a foundation approach for treating hypercholesterolemia, evidenced by the efficient silencing of hepatic transcripts PCSK9 (Lindholm et al. 2012, Rocha et al. 2015, Poelgeest et al. 2015) and ApoB (Crooke et al. 2005, Mullick et al. 2011, Agarwala et al. 2015, Santos et al. 2015). Additionally, α-tocopherol conjugation provides a different pharmacokinetics profile, characterized by an extended half-life compared to unconjugated counterparts (Nishina et al. 2015, Nishina et al. 2008), as well as effective brain delivery of conjugated siRNA via direct intracerebroventricular (ICV) infusion (Uno et al. 2011). More recently, small molecule ligands capable of exploiting endogenous transcytosis pathways across the blood-brain barrier (BBB) have demonstrated considerable promise. For example, BCC10-ASO conjugates leverage γ-secretase mediated transport mechanisms to enhance CNS accumulation, holding potential for therapeutic intervention in neurological disorders (Wang et al. 2025) (Table 1).

Short peptide based CPPs have emerged as another class of ligands for enhancing cellular uptake and intracellular delivery of ONTs (Kristensen et al. 2016). Oligonucleotide analogues, such as peptide nucleic acids (PNAs) and phosphorodiamidate morpholino oligonucleotides (PMOs) have been applied to the CPP conjugates (Bendifallah et al. 2006, Stoodley et al. 2023, Ou et al. 2025). PNAs replace the natural sugar phosphate backbone with neutral *N*-(2-aminoethyl)-glycine units, creating a peptide like structure (Ray & Norden 2000). Similarly, PMOs utilize a morpholine ring in place of the ribose sugar, connected via phosphorodiamidate linkages. These neutral scaffolds help circumvent the charge associated delivery barriers, making them well suited for splice modulation and steric blocking applications (Maksudov et al. 2023). Conjugation to linear polycationic peptides has been utilized to promote membrane association and cellular uptake. For example, octaarginine (R8)-PNA, suppresses microRNA-155 in primary human macrophages and attenuates pro-inflammatory signaling. Furthermore, these conjugates exhibit effective bactericidal activity against *Streptococcus pneumoniae*, the most common cause of community acquired pneumonia. In both in vitro and in vivo insect infection models, these conjugates successfully target the *gyrA* and *rpoB* genes, with anti-*gyrA* PNAs showing superior efficacy (Barkowsky et al. 2022). In addition to linear peptides, conformationally constrained cyclic cell-penetrating peptides (cCPPs) offer enhanced structural rigidity, increased proteolytic resistance, and efficient cytosolic access. For instance, the cytosol localizing internalization peptide (CLIP6)-PNA achieves non-endosomal delivery, increasing bioavailability and cellular uptake. By shifting alternative splicing toward the tumor suppressor Mnk2a and away from the oncogenic Mnk2b variant, this approach induces cell death in glioblastoma models (Soudah et al. 2017). Similarly, cyclic peptide (C9)-PNA targeting miR-155, increases cellular uptake and shows anticancer activity in U87MG glioblastoma cells (Soudah et al. 2019). Moreover, amphipathic cCPPs containing arginine and hydrophobic residues, such as $\:\mathrm{cF}{\Phi\:}{\mathrm{R}}_{4}$ and related analogs, interact directly with membrane phospholipids to enter cells via endocytic pathways, followed by rapid and efficient escape from early endosomes (Qian et al. 2014). Mechanistic investigations demonstrate that these cyclic motifs induce local membrane curvature, triggering the budding and subsequent rupture of endosome derived small vesical (vesicle budding-and-collapse, VBC) to directly release intact cargo into the cytoplasm (Qian et al. 2016). Ongoing chemical optimization of these cyclic scaffolds continues to improve cytosolic delivery efficiency and endosomal escape kinetics, supporting their utility for therapeutic oligonucleotide delivery (Buyanova et al. 2022).

CPP-PMO platforms have been investigated for treating neuromuscular disorders. CPP-PMOs promoting exon 2 skipping leads to widespread dystrophin restoration in the skeletal muscles of Duchenne muscular dystrophy (DMD) models following a single intravenous injection (Gushchina et al. 2022). To further optimize delivery, arginine rich CPPs such as the PNA- and PMO-internalizing peptides Pip6a are frequently employed (Gait et al. 2019). Pip6a-PMO conjugates promote restoration of cardiac dystrophin in DMD mouse models, resulting in marked improvements in heart function (Blain et al. 2018). Pip6a-PMOs effectively correct aberrant splicing in myotonic dystrophy type 1 (DM1). Systemic administration of these conjugates achieves significant therapeutic uptake in skeletal muscle, the heart, and the diaphragm, leading to long lasting normalization of splicing profiles and the reversal of DM1 associated functional defects (Klein et al. 2019). Pip6a-PMOs improve survival and motor function in mouse models of SMA by effectively increasing survival motor neuron (SMN) transcript levels in both the CNS and peripheral tissues (Hammond et al. 2016). Likewise, CCP (DG9)-PMO administered via a single subcutaneous injection extends survival while simultaneously improving muscle strength, respiratory function, and neuromuscular junction integrity in SMA mice (Aslesh et al. 2023). Beyond splice switching ASOs, the versatility of CPPs is further manifested in the delivery of siRNA. A bivalent cyclic RGD peptide facilitates tumor targeted delivery of VEGFR2-siRNA, inhibits VEGFR2 expression and slows tumor progression in a non-small cell lung cancer xenograft model (Liao et al. 2021). In the CNS, peptides derived from rabies virus glycoprotein (RVG) exploit nicotinic acetylcholine receptor (AchR) to enable targeted delivery of siRNA across neural tissues (Oswald et al. 2017, Kumar et al. 2007).

Antibody-oligonucleotide conjugates (AOCs) provide another effective platform for cell specific delivery of ONTs via receptor mediated internalization (Dugal-Tessier et al. 2021). By coupling oligonucleotide to monoclonal antibodies (mAbs) or antibody fragments, these systems exploit high affinity surface receptor recognition to improve issue selectivity and facilitate delivery to extrahepatic sites that remain challenging for conventional chemistries. ASO-based AOCs platforms have proven effective in targeting specific cell populations and overcoming delivery barriers in multiple disease models (Li et al. 2025, Jiao et al. 2024). CD44 mAb-ASOs selectively target the glioblastoma stem cell (GSC) significantly reduces DRR/FAM107A expression and suppresses cell invasiveness in patient derived GSCs (Arnold et al. 2018). Anti-CD22 mAb-ASOs enable B-cell specific silencing of *MXD3*, resulting in robust protein knockdown and extended survival in precursor B cell acute lymphoblastic leukemia (preB-ALL) models (Satake et al. 2016). In addition, anti-transferrin receptor (TfR) antibody-ASOs improved CNS uptake in SMA mice following systemic delivery, leading to splice corrected SMN2 expression and increased survival rates (Hammond et al. 2022). Several other antibodies have been investigated for assisted siRNA delivery. The HIV-1 envelope antibody protamine conjugate (F105-P) enables delivery siRNAs targeting the *gag* gene, resulting in potent inhibition of HIV-1 replication in infected primary T cells. Furthermore, F105-P-siRNA complexes target *c-myc*, *MDM2*, and *VEGF* successfully inhibit envelope protein expression in B16 tumor cells (Song et al. 2005). Similarly, a CD7 antibody fragment conjugated to arginine peptide (scFvCD7-9R) facilitates anti-HIV siRNAs (targeting to *CCR5*,* vif*, and *tat*) delivery to T cells, effectively suppressing HIV infection in humanized mice by blocking viral replication (Kumar et al. 2008). An anti-HER2 antibody fragment protamine fusion (F5-P) conjugated to PLK1-siRNA selectively silences PLK1 expression in HER2^+^ breast cancer cells. This targeted suppression reduces metastasis, induces apoptosis, and prolongs survival without triggering an interferon response. Furthermore, the combined delivery of a siRNA cocktail targeting *PLK1*, *CCND1*, and *AKT* demonstrates superior therapeutic efficiency compared to *PLK1* silencing alone (Yao et al. 2012). Additionally, transferrin receptor (CD71-Fab)-siRNA has achieved sustained gene silencing in cardiac and skeletal muscle of peripheral artery disease (PAD) affected mice, leading to improve muscle performance (Sugo et al. 2016). Driven by their therapeutic possibilities, AOCs in mAb and Fab formats are now being transitioned into clinical trials (Mullard 2022).

Aptamer oligonucleotide conjugates represent a versatile strategy, combining the high affinity molecular recognition of aptamers with potent gene modulation activity of ASOs and siRNA (Zhou and Rossi 2017, White et al. 2000, Sivakumar et al. 2019, Kruspe and Giangrande 2017). By binding cell surface receptors with high affinity, aptamers facilitating receptor mediated uptake, enabling selective delivery and enhanced gene silencing in variety cancer models (McNamara et al. 2006, Nuzzo et al. 2019). Aptamer mediated delivery has been applied to ASOs, splice switching oligonucleotides (SSOs) and siRNA. AS1411-SSO, a chimera comprising a nucleolin targeting aptamer and a SSO, enhances the nuclear delivery of antisense payloads in cancer cells resulting in increased splicing activity in luciferase reporter assays (Kotula et al. 2012). Similarly, aptamer-ASOs utilize caveolae mediated endocytosis to selectively silence *Galectin-1* in tumor cells, a key driver of tumor metastasis when overexpressed (Shanni Hong et al. 2016). Anti-PSMA (prostate specific membrane antigen) aptamer-siRNAs targeting *Lamin A/C*,* PLK1*,* BCL2*, and *EEF2* have been explored for prostate cancer therapy. These conjugates effectively inhibit target gene expression, ultimately triggering apoptosis (McNamara et al. 2006, Chu et al. 2006, Wullner et al. 2008). Bivalent PSMA aptamer constructs fused siRNAs against *EGFR* and *survivin* (Liu et al. 2016). This approach concurrently inhibits both targets, inducing effective apoptosis in PSMA-positive cell lines in vitro, as well as in C4-2 PCa xenograft mouse models in vivo. Aptamer-Gint4.T/STAT3-siRNAs targeting PDGFRβ glioblastoma cells by silencing *STAT3*, a key genetic driver of the disease, it impairs tumor cell survival (Esposito et al. 2018). The H2EH3 trivalent chimera (HER2 aptamer–EGFR siRNA–HER3 aptamer) effectively suppresses tumor progression through co-targeting, offering a potent therapeutic effect and a low immunogenicity profile (Yu et al. 2018). Two nucleolin aptamer–siRNA (aptNCL-SLUGsiR and aptNCL-NRP1siR) chimeras inhibit lung cancer metastasis through the simultaneous downregulation of *SLUG* and *NRP1*, thereby effectively block the key pro-metastatic signaling pathways (Lai et al. 2014). Aptamer-siRNAs have also displayed antiviral potential. The gp120 aptamer–HIV tat/rev siRNAs provide sustained viral suppression in humanized RAG-hu mice, which mimic features of clinical HIV-1 pathogenesis and helper T-cell loss (Neff et al. 2011). A DNA aptamer–siRNA targets HIV-1 protease expression in CD4^+^ T cells, yielding enhanced stability over RNA-aptamers while maintaining potent suppression of viral mRNA (Zhu et al. 2012).

### Clinical translation

The clinical translation of ONTs is increasingly defined by the transition of advanced conjugation strategies from the bench to the bedside. The following section focuses on those that have reached regulatory approval or advanced into clinical trials. Early studies of unconjugated ASOs established the clinical feasibility of sequence specific RNA therapeutics across multiple genetic disorders. DMD stems from mutation within the DMD locus that disrupts the reading frame, resulting in a loss of functional dystrophin leading to progressive muscle wasting (Duan et al. 2021). Current therapies employ unconjugated exon-skipping ASOs such as golodirsen & viltolarsen (targeting exon 53) (Heo and Golodirsen 2020, Dhillon and Viltolarsen 2020, Clemens et al. 2020), casimersen (targeting exon 45) (Shirley and Casimersen 2021, Wagner et al. 2021) and eteplirsen (targeting exon 51) (Syed and Eteplirsen 2016, Cirak et al. 2011, Mendell et al. 2013) reprogram transcript splicing to restore a functional dystrophin protein. Similarly, SMA arises from insufficient functional SMN protein causing motor neuron degeneration and subsequent muscle atrophy (Kolb and Kissel 2015), a deficit addressed by the splice modulating ASO nusinersen, which promotes *SMN2* exon 7 retention to restore therapeutic levels of the intact SMN protein production (Hoy and Nusinersen 2017, Finkel et al. 2017). In amyotrophic lateral sclerosis (ALS), disease associated *SOD1* mutations drive motor neuron loss and muscle paralysis (Kaur et al. 2016). This is counteracted by tofersen, an ASO that targets *SOD1* mRNA for degradation to reduce the expression of the toxic protein (Blair and Tofersen 2023, Miller et al. 2022). Finally, hATTR involves systemic amyloid deposition driven by misfolded mutant TTR (Benson et al. 2018). This is managed by unconjugated ASOs like inotersen which bind and degrade *TTR* mRNA. effectively lower circulating protein levels and minimizing further amyloid deposition (Benson et al. 2018, Keam and Inotersen 2018). Together, these therapies established the therapeutic potential of ASO mediated splice modulation and RNase H-dependent gene silencing, laying the clinical foundation for the development of conjugated oligonucleotide therapeutics with improved tissue tropism and intracellular delivery.

**Table 1:** Overview of conjugation strategies for oligonucleotide therapeutics

Conjugation Strategy	Representative Ligand / Platform	Targeting / Delivery Mechanism	Linker Type	Principal Tissue / Cell Targets	Representative Applications / Therapeutics	Translational Status	Ref.
Small-molecule conjugates	Triantennary GalNAc	ASGPR-mediated endocytosis	Stable amide	Hepatocytes (liver)	siRNAs: givosiran, inclisiran, vutrisiran, nedosiran ASOs: eplontersen, donidalorsen; pelacarsen (Phase III)	FDA-approved; Phase III	(Debacker et al. 2020, Das et al. 2025, Matsuda et al. 2015, Ramirez-Cortes and Menova 2025, Coelho et al. 2023, Riedl et al. 2024, Riedl et al. 2024, Assessing, Keam and Vutrisiran 2022, Syed and Givosiran 2021, Lamb and Inclisiran 2021, Scott et al. 2021, Syed and Nedosiran 2023, Springer and Dowdy 2018, Prakash et al. 2014, Huang 2017, Dhara et al. 2025, Scott and Givosiran 2020, Nie and Eplontersen 2024)
	C16-palmitoyl	Albumin binding and caveolin-dependent endocytosis	Amide or phosphodiester	Central nervous system (CNS), skeletal muscle	Mivelsiran (ALN-APP)	Phase II	(Chappell et al. 2020, Prakash et al. 2019, Brown et al. 2022, Phase)
	Cholesterol & α-tocopherol	Lipoprotein (HDL/LDL) binding and SR-BI-mediated uptake	Phosphodiester, ester, or disulfide	Liver (hepatocytes), skeletal muscle, CNS	Lipophilic ApoB and PCSK9 conjugates	Preclinical	(Raouane et al. 2012, Biscans et al. 2019, Ostergaard et al. 2019, Nishina et al. 2015, Nishina et al. 2008, Uno et al. 2011)
	BCC10	γ-secretase-mediated BBB transcytosis	Stable amide or PEG spacer	Central nervous system (CNS)	BCC10-ASO	Preclinical	(Wang et al. 2025)
Cell-penetrating peptides (CPPs)	Linear CPPs (Pip6a, R8)	Direct membrane interaction and endocytosis	Amide or thioether	Skeletal/cardiac muscle, CNS	Pip6a-PMO, R8-PNA	Preclinical	(Bendifallah et al. 2006, Barkowsky et al. 2022, Gushchina et al. 2022, Gait et al. 2019, Blain et al. 2018, Klein et al. 2019, Hammond et al. 2016, Aslesh et al. 2023)
	Cyclic CPPs / EEVs (cFΦR4, ENTR-EEV)	Vesicle budding-and-collapse (VBC) and direct membrane translocation	Amide, thioether, or disulfide	Skeletal and cardiac muscle	ENTR-601-44 (EEV-PMO for DMD)	Phase I/II	(Soudah et al. 2019, Qian et al. 2014, Qian et al. 2016, Buyanova et al. 2022, A study in 2026, Li et al. 2023)
Antibody & protein scaffold conjugates	Anti-TfR1 (CD71) mAbs / Fab	TfR1-mediated internalization and transcytosis	Cleavable peptide (Val-Cit) or maleimide	Skeletal and cardiac muscle, CNS	DYNE-251 (TfR1-PMO); AOC-1001 (TfR1-siRNA)	Phase III	(Hammond et al. 2022, Sugo et al. 2016, Mullard 2022, ClinicalTrials.gov and Safety, Study)
	Centyrin scaffolds	Receptor-mediated internalization via engineered protein scaffold	Peptide linker or amide	Skeletal muscle, extrahepatic tissues	ABX1100 (centyrin-siRNA for Pompe disease)	Phase I	(Clinical trial ID NCT06109948)
Aptamer-oligonucleotide conjugates	PSMA & nucleolin (AS1411) aptamers	High-affinity aptamer receptor recognition and internalization	Direct phosphodiester extension or sticky ends	Tumors (prostate, lung, glioblastoma)	PSMA-siRNA, AS1411-SSO	Preclinical	(McNamara et al. 2006, Kotula et al. 2012, Chu et al. 2006, Wullner et al. 2008, Liu et al. 2016)

GalNAc conjugation serves as a receptor targeted delivery platform for ONTs. By leveraging high avidity binding to the ASGPR, this strategy overcomes the inefficient uptake of unconjugated oligonucleotides, enabling subcutaneous administration and potent hepatic gene silencing. This platform has enabled multiple FDA-approved or late-stage ASO therapeutics such as eplontersen, donidalorsen and pelacarsen, and siRNA therapeutics, including givosiran, lumasiran, inclisiran, vutrisiran, and nedosiran (Cronin and Yu 2025, Ramirez-Cortes and Menova 2025). Among ASO modalities, GalNAc conjugation facilitates RNase H-mediated mRNA degradation at substantially lower doses than unconjugated ASO. Eplontersen, an approved GalNAc-ASO, builds upon the clinical precedence of inotersen by integrating GalNAc mediated hepatocyte targeting to improve hepatic delivery while maintaining the same RNase H-dependent TTR silencing mechanism (Coelho et al. 2023). Hereditary angioedema (HAE) results from excessive plasma prekallikrein activity, leading to bradykinin mediated swelling attacks. Donidalorsen, an approved GalNAc-ASO that targets *KLKB1* mRNA, reducing hepatic prekallikrein synthesis and preventing recurrent HAE attacks (Riedl et al. 2024, Riedl et al. 2024). Cardiovascular disease associated with elevated lipoprotein(a) is driven by hepatic production of apolipoprotein(a). Pelacarsen (TQJ230), currently in Phase III development, employs GalNAc-ASO mediated hepatocyte targeting to silence *LPA* mRNA, thereby lowering circulating lipoprotein(a) levels (Assessing).

In addition to ASO therapeutics, GalNAc-mediated delivery has similarly enabled the clinical translation of siRNA therapeutics through efficient hepatocyte specific RNA interference (RNAi). Vutrisiran indicated for adult ATTR amyloidosis, leverages the RNAi pathway to degrade *TTR* mRNA, effectively reducing serum mutant TTR protein levels (Keam and Vutrisiran 2022). Givosiran targets δ-aminolevulinic acid synthase 1 (*ALAS1*) mRNA to mitigate the buildup of neurotoxic heme intermediates, thereby decreasing the incidence of composite porphyria attacks in patients with acute hepatic porphyria (AHP) (Syed and Givosiran 2021). Inclisiran, an adjunct to dietary therapy for hypercholesterolemia, inhibits PCSK9, increasing LDL receptor expression and promoting clearance of LDL cholesterol (Lamb and Inclisiran 2021). Approved for primary hyperoxaluria type 1, lumasiran reduces oxalate production by targeting *HAO1* mRNA, which encodes glycolate oxidase, whereas nedosiran lowers oxalate by targeting hepatic *LDH* mRNA. Both therapies effectively decrease urinary oxalate levels through distinct points in the oxalate metabolic pathway (Scott et al. 2021, Syed and Nedosiran 2023). Collectively, these GalNAc-conjugated ASO and siRNA therapeutics demonstrate how receptor mediated hepatocyte targeting has addressed key translational hurdles, establishing a practical, systemic therapeutic platform for diverse liver associated diseases.

Advances in GalNAc-mediated delivery have inspired the exploration of complementary platforms to extend the reach of oligonucleotide therapeutics beyond hepatocytes. The current frontier includes diverse conjugation strategies, such as AOCs and other types of ligands, designed to overcome the bioavailability barriers that limit oligonucleotide efficacy in extrahepatic tissues. For example, DYNE-251 in Phase III trials (NCT05524883), is an AOC-PMO that utilizes a TfR1-targeting antibody to deliver a PMO directly to muscle tissue. This targeted approach builds upon established exon skipping therapies for DMD, rescuing reading frame integrity to express a shortened dystrophin product that retains essential mechanical function (ClinicalTrials.gov and Safety). Cyclic peptide-mediated PMO delivery has also advanced into clinical development. ENTR-601-44, an exon 44-skipping PMO conjugate is currently being evaluated in the Phase 1/2 ELEVATE-44-201 study (NCT07037862) in DMD patients with mutations correctable by exon 44 exclusion. Initial clinical results showed exon 44 skipping and increased dystrophin production, highlighting the translational potential of this strategy (A study in 2026, Li et al. 2023).

siRNA based AOC-1001 utilizes same mechanism to degrade mutant *DMPK* mRNA and preventing MBNL sequestration, this Phase III (NCT06411288) candidate seeks to improve muscle function in patients with DM1 (ClinicalTrials.gov 2026). Currently in Phase II trials (NCT06393712), mivelsiran (ALN-APP) is a C16-conjugated siRNA designed to degrade amyloid precursor protein (*APP*) mRNA and reduce the accumulation of toxic amyloid plaques in patients with cerebral amyloid angiopathy (ClinicalTrials.gov 2025). ABX1100 is an engineered protein scaffold centyrin conjugated siRNA therapeutic in Phase I trial (NCT06109948). It targets *GYS1* mRNA to mitigate pathological glycogen accumulation in Pompe disease (ClinicalTrials.gov 2026). The strategic application of conjugation in oligonucleotide therapeutics, as evidenced by this expanding clinical portfolio, reflects a broader evolution in drug development and the successful translation of genomic medicine from laboratory to clinic.

### Future outlook

Substantial progress in oligonucleotide chemistry and targeted delivery has enhanced both pharmacological activity and therapeutic precision. This momentum is increasingly driven by ligand functionalized ASO and siRNA platforms, which have broadened the clinical utility of treatments for previously intractable diseases. Although GalNAc-mediated delivery has driven clinical advancements in multiple indications, this platform has several aspects for consideration, including hepatocyte tropism and receptor saturation at high exposure levels (Dowdy 2017, Springer and Dowdy 2018, Prakash et al. 2014). Furthermore, delivery efficiency relies heavily on ligand valency, linker, and spatial arrangement. Studies on mono-, bi-, tri-, and tetra-antennary GalNAc conjugates show that optimized ligand density and orientation are vital for maximizing receptor binding, cellular uptake, and in vivo silencing efficacy, with the triantennary configuration remaining the most clinically established and preferred format (Liu et al. 2025, Huang 2017, Dhara et al. 2025). Together with emerging approaches, these insights are driving the expansion of therapeutic oligonucleotides into extrahepatic tissues, such as the CNS and skeletal muscle.

Despite advancements in ONTs, several challenges need to be addressed for broad clinical applicability. A primary rate-limiting barrier to clinical translation remains efficient and specific tissue targeting (Gokirmak et al. 2021, Hammond et al. 2021). Conjugates must achieve selective delivery to the intended tissue and cell type while minimizing accumulation in off-target organs. Following cellular uptake, endosomal escape presents an additional major challenge, as only a small fraction of the internalized payload reaches the cytosol or nucleus, where therapeutic activity occurs (Allen and Yokota 2024, Dowdy 2023, Grau and Wagner 2024). Addressing these barriers is a central focus of current research, with emerging strategies including the identification of tissue and cell specific ligands, pH-responsive and cleavable linkers, ionizable chemistries, and the co-administration of small molecule escape enhancers (Allen and Yokota 2024, Mangla et al. 2023, Juliano 2021). Future efforts are also exploring mechanisms that bypass the endosome entirely through direct plasma membrane translocation or exploit endogenous transcytosis pathways for cargo delivery (Gokirmak et al. 2021, Nagaraj et al. 2025).

In parallel to these delivery challenges, safety remains critical consideration. Conjugated ONTs demand rigorous evaluation of immunogenicity, and off-target tissue exposure arising from nonspecific RNA binding. Clinical translation is further challenged by the need for scalable manufacturing of highly modified oligonucleotides conjugates with consistent quality and reproducibility. By integrating advancements in chemical design, receptor biology, and translational pharmacology, next-generation conjugates are primed to bridge the gap between bench to personalized therapy, ultimately expanding the therapeutic scope of genomic medicine.

## Data Availability

Not applicable (no datasets were generated or analyzed).
